# The effects of SNAP emergency allotments on state-level SNAP benefits and enrollment during the COVID-19 pandemic

**DOI:** 10.1093/haschl/qxae109

**Published:** 2024-08-28

**Authors:** David R Steffen, David D Kim

**Affiliations:** Harris School of Public Policy, University of Chicago, Chicago, IL 60637, United States; Division of Biological Sciences, Department of Medicine and Public Health Sciences, University of Chicago, Chicago, IL 60637, United States

**Keywords:** SNAP, COVID-19, program evaluation, health policy, difference-in-difference

## Abstract

During the COVID-19 pandemic, all US states provided emergency allotments (EA) to enrollees of the Supplemental Nutrition Assistance Program (SNAP) to alleviate rising food insecurity. However, 18 states opted out of the SNAP-EA program before its official expiration in February 2023. Using a staggered difference-in-differences model to account for state-level variation in the timing of the SNAP-EA opt-out decisions, we analyzed SNAP and SNAP-EA data from the US Department of Agriculture Food and Nutrition Service to quantify the impact of state opt-out decisions on SNAP benefit size and enrollment. We found that the average SNAP monthly benefit among 18 SNAP opt-out states was reduced by $183 (95% confidence interval [CI]: −$214, −$152) per beneficiary. The percentage of the state population enrolled in the SNAP program among the opt-out states modestly decreased by 0.35 (95% CI: −0.61, −0.10) percentage points. Additionally, we employed logistic regression models to associate state opt-out decisions with state-level characteristics. We found that the state governor's political party being Republican was the only significant predictor for the state's opt-out decisions. Our findings help explain why opting out of SNAP-EA has been associated with higher food insufficiency and shed light on the impact of political decisions to opt out of SNAP-EA on the lives of millions of Americans.

## Introduction

In March 2020, the US Department of Agriculture (USDA)—the federal agency that administers the Supplemental Nutrition Assistance Program (SNAP)—provided emergency allotments (EA) to alleviate rising food insecurity during the COVID-19 pandemic. Under the Families First Coronavirus Response Act,^1^ the SNAP-EA program provides all households an additional $95 or up to the maximum monthly benefit for their household size, whichever is greater.^2^

Until the official expiration of SNAP-EA in February 2023 under the Consolidated Appropriations Act,^3^ state SNAP agencies could continue to provide monthly SNAP-EA to all households as long as national and state Public Health Emergency declarations were in place. Some states requested extensions for the continued issuance of SNAP-EA, which the USDA granted. In contrast, other states ended the SNAP-EA program earlier (eg, Idaho was the first state to terminate the SNAP-EA in April 2021). By February 2023, 18 states had revoked SNAP-EA, while the remaining 32 and Washington, DC, still had it.

Some studies have examined the impact of states opting out of SNAP-EA on food insufficiency, primarily using US Census Bureau Household Pulse Survey data. These studies varied in the study period analyzed and their statistical methods, but they primarily found that opting out of SNAP-EA was associated with higher food insufficiency.^4-7^ However, these studies did not investigate the pathway by which opting out of SNAP-EA impacted food insufficiency, such as the impact of opting out of SNAP-EA on the SNAP program benefits and enrollment. One report compared the average SNAP benefit size before and after the overall end of the SNAP-EA program, but did not investigate state-level variation in SNAP-EA opt-out timing.^8^

Consequently, much remains unknown about the effects of the state-level variations in the timing of the SNAP-EA program termination on state-level SNAP benefits and enrollment during the COVID-19 pandemic. This study aims to estimate the impact of ending the SNAP-EA program on SNAP benefit size and enrollment during the COVID-19 pandemic using a staggered difference-in-differences design.

## Data and methods

### SNAP and SNAP-EA data on enrollment and benefits

We extracted the USDA Food and Nutrition Service (FNS) SNAP monthly data from March 2019 to September 2023 from the FNS website.^9^ The SNAP data include the number of households receiving SNAP benefits, the total amount of money provided, and the monthly total SNAP benefit per household for each state.

The COVID-19 SNAP-EA program was implemented nationwide in March/April 2020 and expired in February 2023. We further collected data on each state's active participation in the SNAP-EA program in any given month using the FNS data.^10^ In addition, we collected the number of households receiving SNAP-EA benefits and the total amount of money provided each month during the period. Based on this information, we calculated the monthly SNAP-EA benefit per household. We used annual state-level population estimates from the Census Bureau to determine the percentage of households in each state receiving SNAP benefits.

### Main analysis: staggered difference-in-differences

To estimate the impact of the SNAP-EA program on benefits and enrollment in the SNAP program, we exploited state-level variation in SNAP-EA opt-out decisions and the timing of SNAP-EA opt-out. We applied the Callaway and Sant’Anna staggered difference-in-differences (CS-DID) approach that accounts for units (in our case, states) being exposed to a treatment (in our case, the policy decisions to opt out of SNAP-EA) at different time points,^11^ using the open-source R package available on GitHub.^12^

The treatment group consisted of the 18 SNAP-EA opt-out states between March 2021 (Idaho) and January 2023 (South Carolina), and the control group included the 32 non-opt-out states and Washington, DC, that never opted out during the study period. Time was measured as the number of months since the beginning of the SNAP-EA program (April 2020, when SNAP-EA was implemented in all states), and the ID variable was each state. The primary outcomes of interest were the average SNAP benefit size and percentage of the state population enrolled in SNAP, calculated as discussed above.

We first measured the average treatment effect of opting out of SNAP-EA across all SNAP-EA opt-out periods as well as the dynamic average treatment effect of opting out of SNAP-EA at each of the cumulative months before or after SNAP-EA opt-out. In addition, we estimated the average treatment effect of opting out of SNAP-EA in each opt-out state (ie, state-specific effects) and the average of group-treatment effects of opting out of SNAP-EA across groups (with groups defined for each month that is the first month of opt-out for at least one opt-out state, eg, March 2021 for Idaho, May 2021 for North Dakota, etc.).

**Figure 1.**
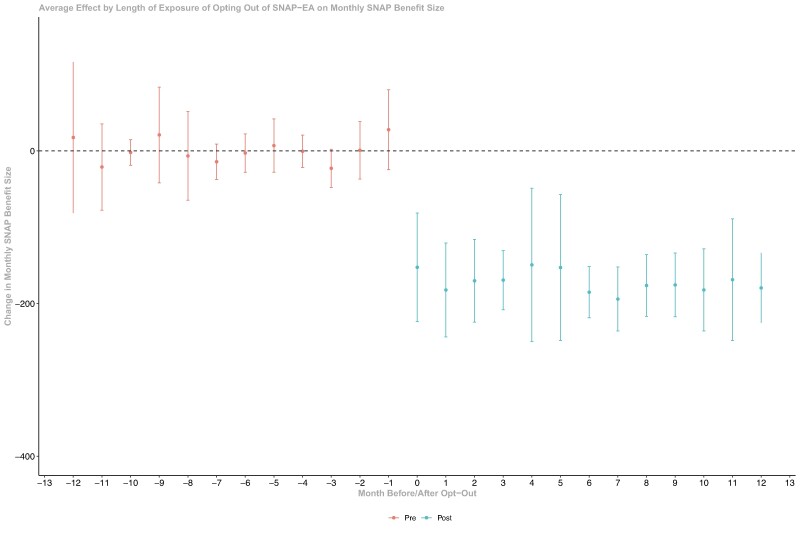
State-level trajectories in SNAP household benefit size after opting out of SNAP-EA. Abbreviations: EA, emergency allotments; SNAP, Supplemental Nutrition Assistance Program.

**Figure 2.**
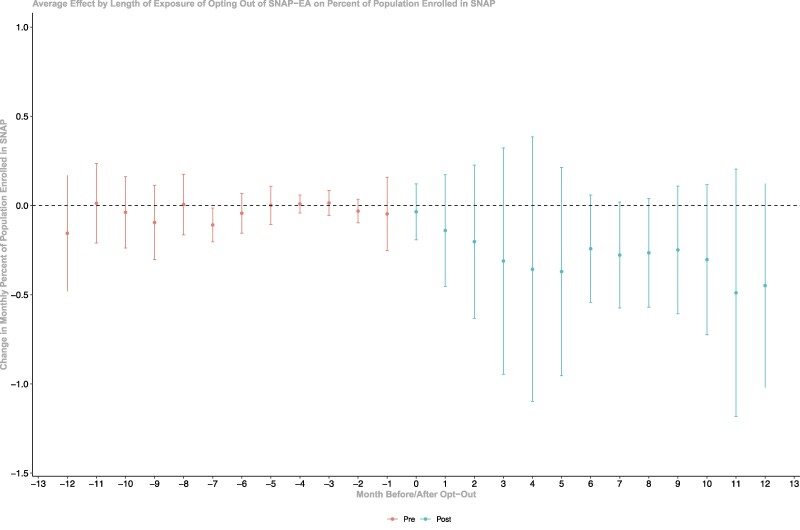
Timeline of state-level SNAP-EA opt-out decisions. Abbreviations: EA, emergency allotments; SNAP, Supplemental Nutrition Assistance Program.

Our analysis satisfies the parallel trends assumption, as there were no statistically significant differences in SNAP benefit size and enrollment prior to the SNAP-EA opt-out between the states that opted out of SNAP-EA and those that did not (Figures 1 and 2).

As sensitivity analyses, we further examined the impact of non-time-varying covariate adjustments for the political affiliation of the state's governor at the time of the SNAP-EA opt-out.

### Secondary analysis: association of state-level characteristics with the opt-out decisions

We further employed logistic regression models to associate state opt-out decisions with state-specific characteristics. We included the state governor's political party affiliation, population size, and unemployment rate from the University of Kentucky Center for Poverty Research National Welfare Data,^13^ 2022 Cook Partisan Voting Index (PVI),^14^ and the SNAP Policy Index. The SNAP policy index, developed by Stacy et al., is a composite measure of specific policies that are likely to encourage SNAP participation.^15^ The SNAP index ranges from 1 (less accommodating) to 10 (more accommodating), and we applied a weighted version of the SNAP policy index that weights each policy by its estimated contribution to the SNAP caseload. Sensitivity analyses included alternative model specifications using linear probability models, the political affiliations of the governor and the majority of the state legislature, and the unweighted SNAP policy index.

## Results

### Overall SNAP enrollment and benefits size during the pandemic

The average percentage of the state population enrolled in SNAP increased from 5.7% in the pre-pandemic period (March 2019-February 2020) to 6.3% in the early pandemic (April 2020-March 2021) in each month averaged across all US states (Figure S1). The average percentage of the state population receiving SNAP-EA among the population receiving any SNAP benefits increased from 59.3% during the early pandemic to 83.6% between May 2021 and February 2023 (Figure S2).

The average SNAP benefits in each month across all states increased from $242 in March 2019-February 2020 to $337 in April-December 2020 due to the SNAP-EA program and increased further to $427 in January-March 2021 due to the annual SNAP cost-of-living adjustment in October 2020 (Figure S3). The average monthly SNAP-EA benefit amounts across all states were fairly consistent over the program period, ranging from $170 to $196 from April 2020 to February 2023 (Figure S4).

### Effects of SNAP-EA on state-level SNAP benefits and enrollment

Our CS-DID analysis found that the average SNAP monthly benefit among 18 SNAP opt-out states across all months after the opt-out decisions was reduced by $183 (95% confidence interval [CI]: −$214, −$152). The percentage of the state population enrolled in the SNAP program among the opt-out states modestly decreased by 0.35% (−0.61%, −0.10%) (Table S1) as averaged across all months after opt-out and by 0.32% (−0.53%, −0.12%) when averaged across all months in which a state dropped out.

Dynamic average policy effects of opting out of SNAP-EA among the opt-out states found that the reduced SNAP benefits as a result of opting out of SNAP-EA were immediately observed after states’ decision to opt-out and sustained over the year after opt-out. The average monthly benefits were reduced by $149-$194 over the 12 months after the opt-out decision (Figure 1). The effect on the percentage of the population enrolled was smaller in the first month after opt-out, ranging from −0.14% to −0.49% in each subsequent month in the first 12 months after opt-out, although the month-level average effects were not significant (Figure 2).

### State-specific effects and sensitivity analyses

The opt-out decisions led to statistically significant reductions in benefit size in each of 17 opt-out states, varying from North Dakota (−$282.56; 95% CI: −$325.49, −$239.62) to Arkansas (−$111.17; 95% CI: −$135.32, −$87.02). The only exception was Alaska, which had a significant positive impact (Table 1, Figure S5). This trend in state-specific effects in SNAP benefit size was consistent over time, reporting a decrease in average household SNAP benefits of $160 from the first month after opt-out to $167 in the fourth month after opt-out (Figure S6).

**Table 1.** State-specific effects of opting out of SNAP-EA.

State-specific treatment effects	Average monthly SNAP benefit size	Percentage of state population enrolled in SNAP
Average of state-specific treatment effects	−164.63 (−180.46, −148.81)*	−0.32 (−0.53, −0.12)*
Group average		
Idaho	−220.38 (−234.89, −205.88)*	−0.18 (−0.34, −0.01)*
North Dakota	−282.56 (−325.49, −239.62)*	−0.37 (−0.54, −0.21)*
Arkansas	−111.17 (−135.32, −87.02)*	−0.81 (−0.98, −0.64)*
Florida, Montana, Nebraska	−148.85 (−221.31, −76.39)*	−0.23 (−0.95, 0.49)
Missouri	−156.24 (−177.14, −135.34)*	−0.30 (−0.49, −0.11)*
Mississippi, Tennessee	−179.70 (−200.87, −158.54)*	−0.34 (−0.60, −0.07)*
Iowa	−245.00 (−259.61, −230.38)*	−0.10 (−0.31, 0.12)
Arizona, Kentucky, Wyoming	−196.75 (−232.49, −161.02)*	0.02 (−0.28, 0.32)
Georgia, Indiana	−187.68 (−208.96, −166.39)*	−0.22 (−0.47, 0.04)
Alaska	124.56 (108.01, 141.10)*	−2.21 (−2.34, −2.08)*
South Carolina	−184.31 (−218.54, −150.08)*	0.04 (0.00, 0.08)

*: confidence band of state-specific effect does not cover 0. Abbreviations: EA, emergency allotments; SNAP, Supplemental Nutrition Assistance Program.

The state-specific effects of the SNAP-EA opt-out decisions on the percentage of the population enrolled in SNAP among the opt-out states were mostly negative, yet statistically insignificant, except in South Carolina, which showed a minuscule increase (0.04 percentage points [0.00, 0.08]) in the SNAP enrollment (Table 1, Figure S7). This trend in state-specific effects in SNAP enrollment was also consistent over time, with states reporting small but gradual decreases in average SNAP enrollment from 1.5 percentage points lower in the first month to 4.7 percentage points lower in the fourth month after opt-out (Figure S8).

Our sensitivity analysis, which adjusted for the political affiliation of the state's governor at the time of SNAP-EA opt-out, did not change our main results (Table S2).

### Characteristics of the opt-out states

Of the 18 opt-out states, 17 had Republican governors at the time of the end of their declaration of emergency and subsequent SNAP-EA opt-out. Additionally, 16 of the 18 opt-out states voted for the Republican candidate in the 2020 presidential election, and the 18 opt-out states include 14 of the 20 most Republican states as defined by the 2022 Cook PVI.^14^ In multivariable regression analyses adjusting for 2022 PVI, 2019 population, 2019 unemployment rate, and weighted SNAP Policy Index in April 2020 (Table S3), the state governor's political party affiliation was the only significant predictor. The marginal effect of having a Republican governor on the probability of a state opting out of the SNAP-EA program was 0.332 (95% CI: 0.057, 0.608). In multiple sensitivity analyses, the result remained consistent (Table 2).

**Table 2.** State-level opt-out regressions.

	Model 1 (Republican governor 2021-2023)			Model 2 (Republican state legislature 2021-2023)			Model 3 (Republican governor and state legislature 2021-2023)		
	LPM	Probit	Logit	LPM	Probit	Logit	LPM	Probit	Logit
R governor	0.3820** (0.1336, 0.6305)	0.3407* (0.0664, 0.6150)	0.3321* **(0.0565, 0.6077)**	—	—	—	—	—	—
R legislature	—	—	**—**	0.4640* (0.1126, 0.8153)	0.5432** (0.2102, 0.8761)	0.5528*** (0.2162, 0.8894)	—	—	—
R governor and legislature	—	—	**—**	—	—	—	0.5123*** (0.2364, 0.7881)	0.4251* (0.0393, 0.8109)	0.4089* (0.0316, 0.7863)
2022 PVI	0.0098 (0.0001, 0.0194)	0.0100* (−0.0077, 0.0186)	**0.0101** **(−0.0040, 0.0243)**	0.0003 (−0.0140, 0.0147)	−0.0015 (−0.0194, 0.0163)	−0.0018 (−0.0193, 0.0158)	0.0034 (−0.0073, 0.0140)	0.0055 (−0.0077, 0.0186)	0.0058 (−0.0087, 0.0203)
Population (2019, 100s of thousands)	−0.0005 (−0.0019, 0.0009)	−0.0006 (−0.0020, 0.0009)	**−0.0006** **(−0.0022, 0.0010)**	−0.0009 (−0.0024, 0.0005)	−0.0014 (−0.0030, 0.0002)	−0.0014 (−0.0036, 0.0008)	−0.0006 (−0.0020, 0.0007)	−0.0007 (−0.0022, 0.0008)	−0.0007 (−0.0024, 0.0009)
Unemployment rate (2019)	0.0630 (−0.0617, 0.1878)	0.0410 (−0.0928, 0.1748)	**0.0420** **(−0.1091, 0.1931)**	−0.0133 (−0.1433, 0.1166)	−0.0217 (−0.1453, 0.1018)	−0.0312 (−0.1637, 0.1014)	0.0439 (−0.0746, 0.1625)	0.0496 (−0.0874, 0.1866)	0.0504 (−0.1068, 0.2075)
SNAP Policy Index (weighted, April 2020)	−0.0610 (−0.1294, 0.0073)	−0.0557 (−0.1137, 0.0023)	**−0.0599** **(−0.1441, 0.0243)**	−0.0830* (−0.1496, −0.0165)	−0.0651** (−0.1106, −0.0196)	−0.0687 (−0.1573, 0.0199)	−0.0532 (−0.1191, 0.0128)	−0.0506 (−0.1097, 0.0084)	−0.0545 (−0.1364, 0.0274)
*N*	51	51	**51**	50	50	50	50	50	50
AIC	47.9013	44.4442	**44.7455**	49.4898	43.0050	42.5193	43.4148	42.9448	43.3661
BIC	61.4241	56.0351	**56.3365**	62.8740	54.4771	53.9914	56.7989	54.4169	54.8383

Bold text: primary analysis. *** *P* < 0.001; ** *P* < 0.01; * *P* < 0.05. Abbreviations: AIC, Akaike information criterion; BIC, Bayesian information criterion; EA, emergency allotments; LPM, linear probability model; PVI, Partisan Voting Index; SNAP, Supplemental Nutrition Assistance Program.

## Discussion

During the early COVID-19 pandemic, the population enrolled in SNAP increased, possibly due to increased unemployment and poverty. Despite the availability of the SNAP-EA program, which provided additional SNAP benefits to each household in each month from 2020 to 2023, 18 states chose to end their state declarations of emergency before the end of the national declaration of emergency. As a result, those states had to opt out of federal funding for the SNAP-EA program.

The state-level decisions to leave the SNAP-EA program led to small but gradual decreases in average SNAP enrollment among opt-out states, averaging −0.35 percentage points across all months after opt-out. While opt-out states reported a lower percentage of their population enrolled in SNAP than non-opt-out states, this trend was observed even before these states opted out. Also, a lower proportion of SNAP enrollees in the opt-out states remained largely unchanged after the opt-out decisions.

Also, SNAP benefits in opt-out states decreased by an average of $182 per household averaged across the month after opt-out. These effects were largely consistent across the 12 months following opt-out decisions (dynamic average policy effects). They were mostly consistent across almost all opt-out states in state-specific analyses by each month in which states opted out of the SNAP-EA.

The exception to the decrease in SNAP benefit size was Alaska, which reported an increase in monthly benefit size in the immediate months after opt-out and had the largest effect of opting out on the percentage of the population enrolled in SNAP. We believe that a decrease in the number of beneficiaries (denominator) after the coincident reinstatement of recertification requirements led to an increase in the average SNAP benefits per person after the opt-out decision and a larger decrease in the percentage of the population enrolled than in other opt-out states, despite the reductions in total SNAP benefits (numerator). Another study found a similar impact of SNAP-EA in Alaska.^4^

Building upon prior literature highlighting that opt-out states were associated with higher food insufficiency,^4-7^ our study identified that the immediate reduction in the size of SNAP benefits—which was consistently observed among opt-out states—would be a primary mechanism through which opting out of SNAP-EA impacted the lives of millions of Americans.

Governors usually made decisions to end declarations of emergency. Of the 18 opt-out states, 17 were led by Republican governors, and those states were predominantly among the most Republican-leaning in the country. The sole exception was Kentucky, where the declaration of emergency was ended by legislation passed by a Republican state legislature. Indeed, Democratic Governor Andy Beshear explicitly referenced that ending the state declaration of emergency would end Kentucky's SNAP-EA funding and leave “more than half a million Kentuckians—including Kentucky children—without the emergency assistance they need to buy healthy food for their families” as the primary reason for his veto,^16^ which was overridden by a state legislature supermajority. This underscores that the potential impact of opting out of SNAP-EA on the size and accessibility of SNAP benefits was well understood by the political actors who were making decisions on whether to opt out of the program or not.

Our study has some limitations. First, we did not account for differential timing in approving and issuing SNAP benefits in a given month. However, our consistent findings across dynamic average policy effects, state-specific analysis, and calendar-month-specific analysis showed that our main findings were robust. Second, there were a small number of months where SNAP-EA data were missing or inconsistent. However, the overall SNAP program data used in these analyses were available in all months in all states except for one month in Wyoming. Third, our use of administrative SNAP data did not enable the analysis of the reasons for state-level SNAP-EA opt-out decisions or the impact of reduced SNAP benefits on participant health outcomes, which could be areas for future study. Finally, in our analysis to examine state-level characteristics associated with the opt-out decisions, we used some data inputs collected in pre-pandemic (or early-pandemic) periods due to the lack of availability of more recent data. Although it is unlikely for these variables to change drastically in recent years among opt-out states, further analyses could be performed by adjusting for time-concordant state-specific data.

## Conclusion

The policy design of the SNAP-EA program required states to stop receiving additional SNAP benefits if they ended their state declarations of emergency. That design choice, combined with the decisions by 18 state governments to end their declarations of emergency, meant that millions of SNAP recipients eventually received hundreds of dollars less per month in SNAP benefits than those in states that did not opt out of the program, and fewer of the residents of those states received SNAP benefits than of the residents in states that did not opt out of the program. Our study could shed light on the profound impact of political decisions to end COVID-era emergency declarations on the lives and health of millions of vulnerable Americans experiencing economic precarity.

## Supplementary Material

qxae109_Supplementary_Data
