# Supplementary material for: The effects of SNAP emergency allotments on state-level SNAP benefits and enrollment during the COVID-19 pandemic
Source: Health Aff Sch. 2024 Aug 28;2(9):qxae109. doi: 10.1093/haschl/qxae109 (PMC11426163; doi:10.1093/haschl/qxae109)
Supplement: qxae109_Supplementary_Data [file qxae109_supplementary_data.zip › HASCHOLAR-D-24-00138.R1 - Online Appendix.pdf]

SUPPLEMENTAL MATERIAL

Supplemental Figure 1. Monthly Percentage of State Populations Receiving SNAP-EA

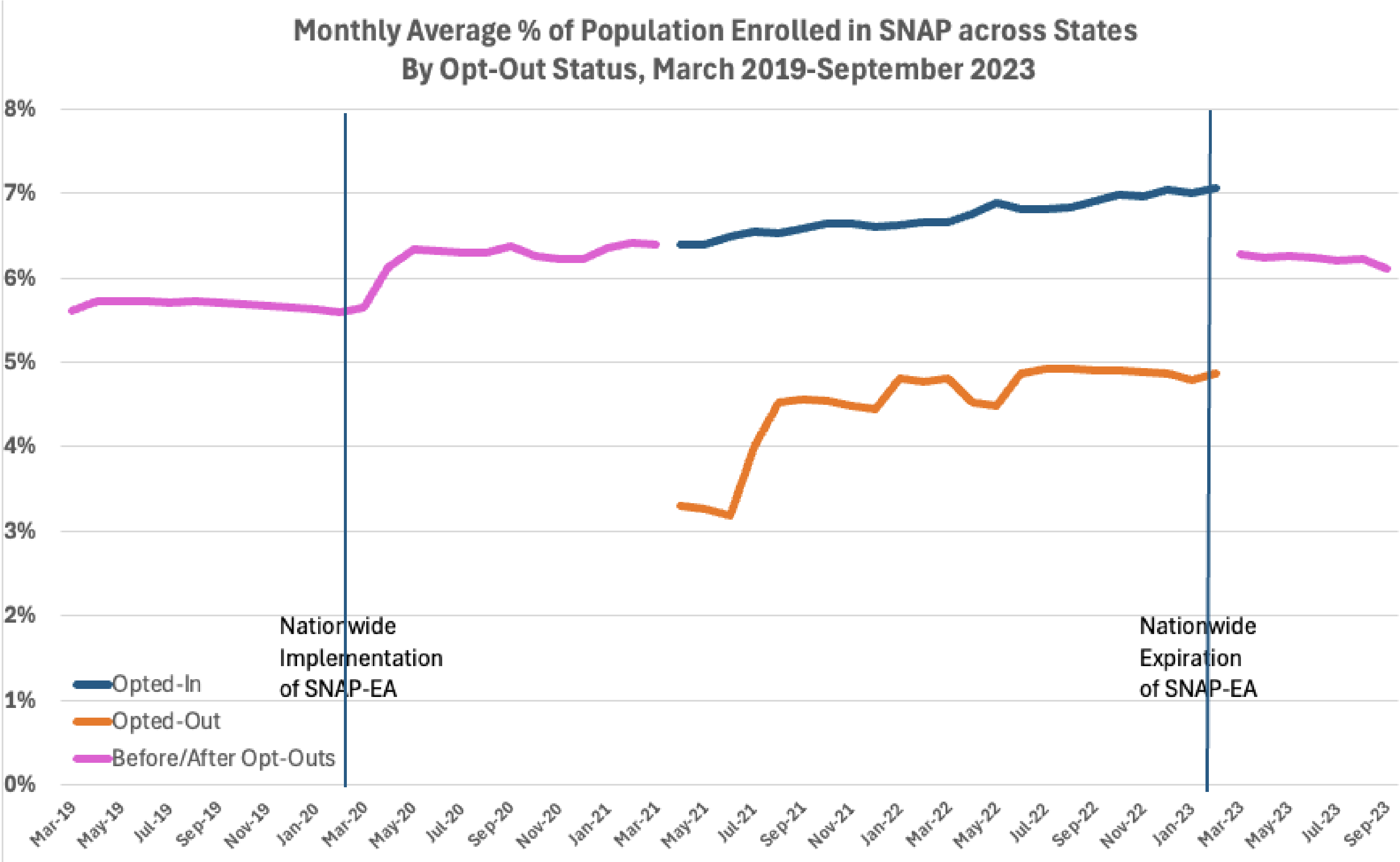

Supplemental Figure 2. Monthly Average Percentage of Population Enrolled in SNAP-EA across States

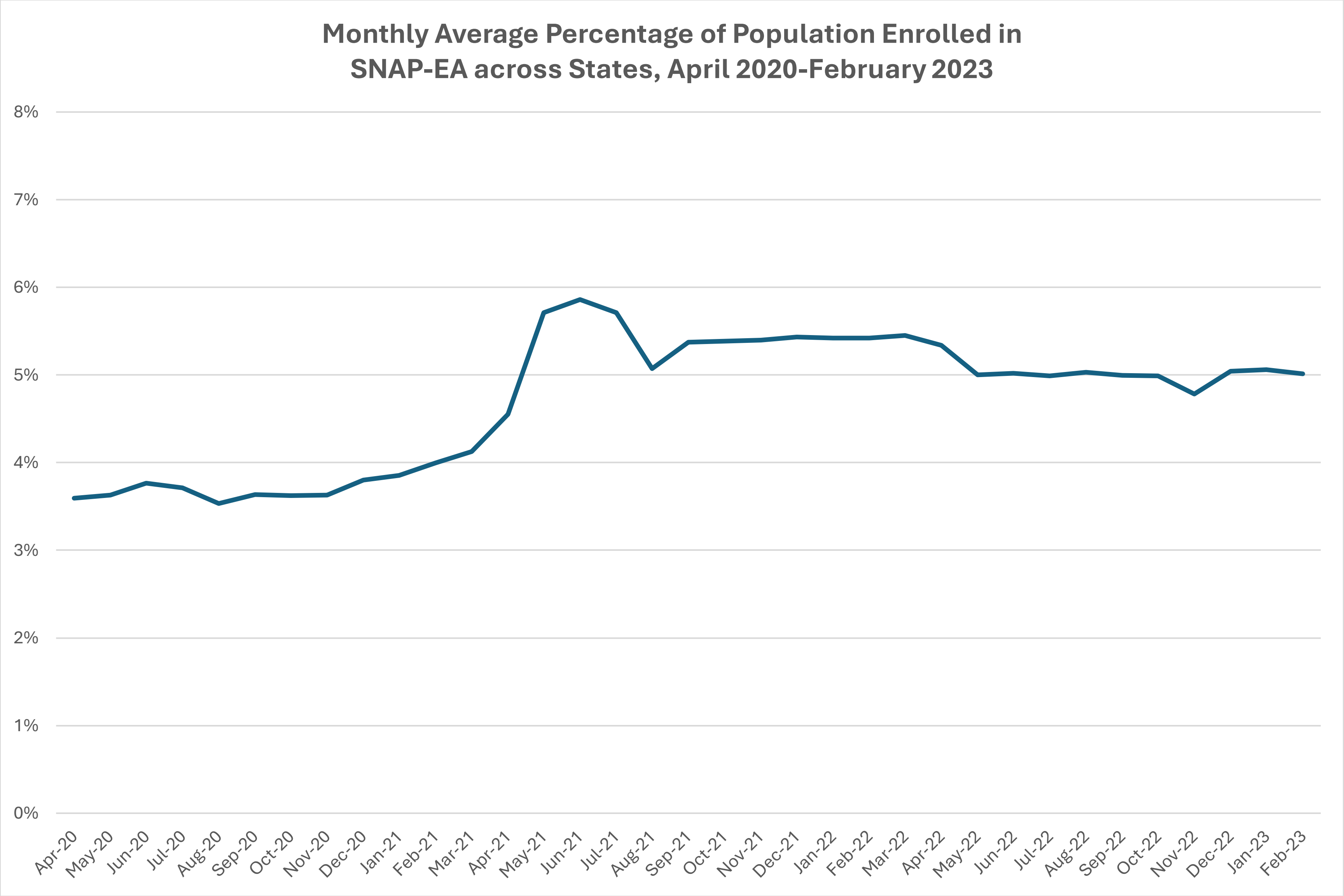

Supplemental Figure 3. Monthly Average SNAP Benefit Amount across States by Opt-Out Status

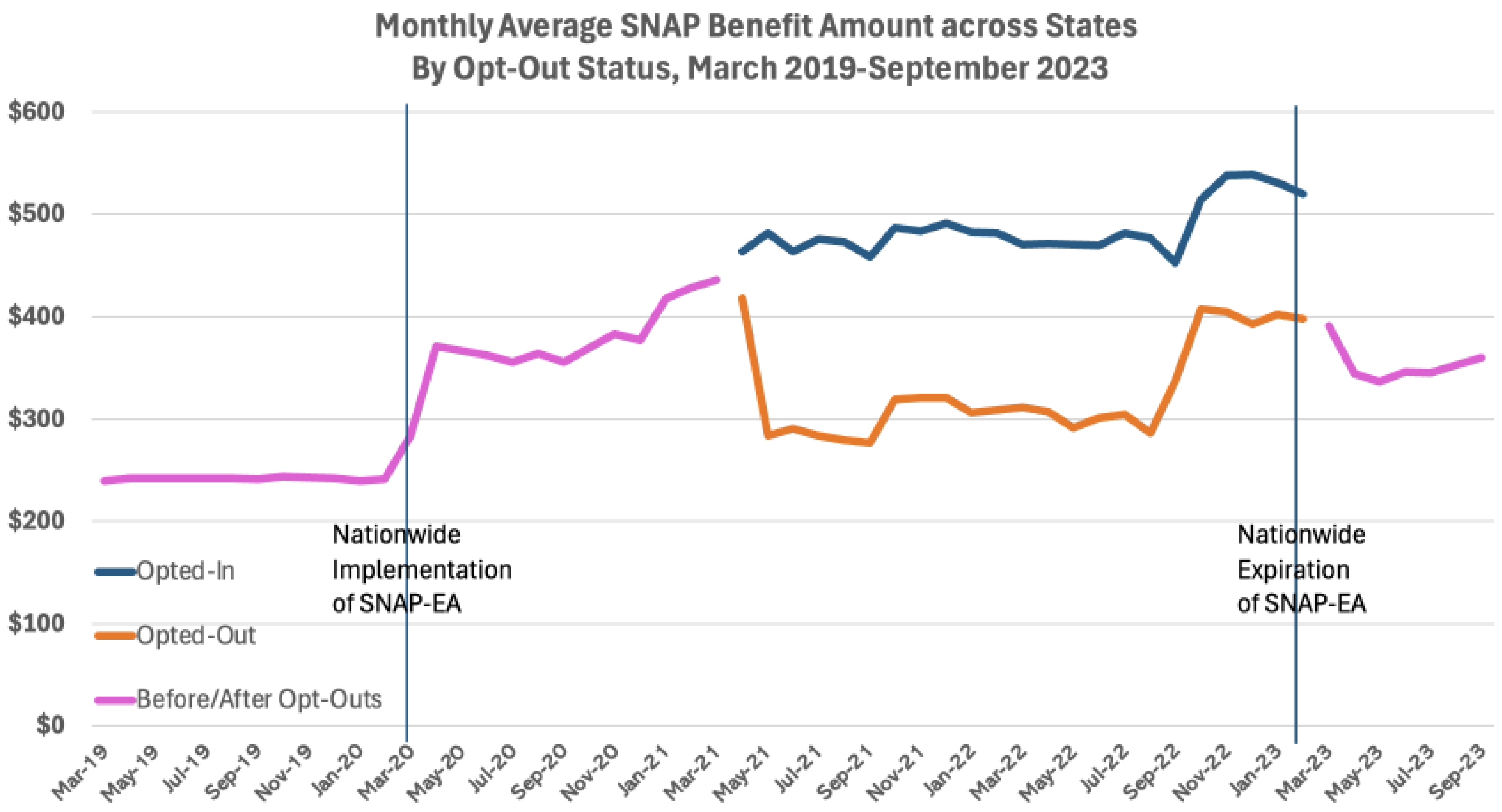

Supplemental Figure 4. Monthly Average SNAP-EA Benefit Amount across States

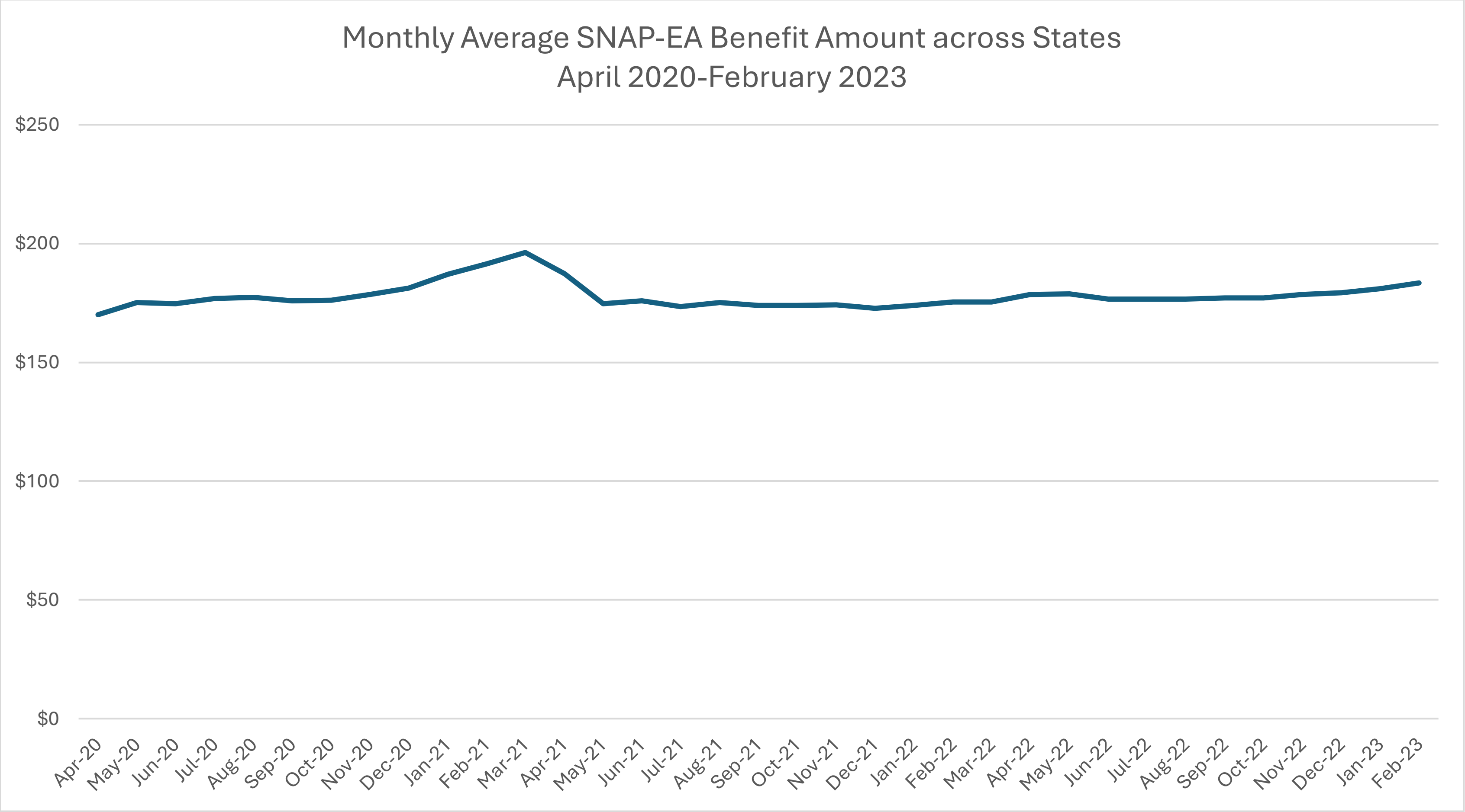

Supplemental Table 1. Average Effects of opting out of SNAP-EA by months before and after opt-out

Average treatment effects	Average monthly SNAP benefit size	Percentage of state population enrolled in SNAP
**Group-time aggregate across all lengths of exposure**	-182.80 [-213.83, -151.77] *	-0.35 [-0.61, -0.10] *
**Average Effect by Length of Exposure**		
-12	17.48 [-75.83, 110.79]	-0.16 [-0.49, 0.18]
-11	-21.18 [-76.51, 34.15]	0.01 [-0.21, 0.23]
-10	-2.14 [-19.97, 15.68]	-0.04 [-0.24, 0.16]
-9	20.86 [-37.8, 79.53]	-0.10 [-0.30, 0.11]
-8	-6.64 [-65.29, 52.00]	0.01 [-0.17, 0.18]
-7	-14.30 [-37.46, 8.87]	-0.11 [-0.21, -0.01] *
-6	-2.89 [-25.15, 19.37]	-0.04 [-0.15, 0.07]
-5	6.79 [-29.84, 43.42]	0.00 [-0.11, 0.11]
-4	-0.59 [-21.97, 20.78]	0.01 [-0.04, 0.06]
-3	-22.92 [-51.00, 5.16]	0.01 [-0.05, 0.08]
-2	0.74 [-37.76, 39.23]	-0.03 [-0.10, 0.03]
-1	27.67 [-25.87, 81.20]	-0.05 [-0.24, 0.15]
0	-152.53 [-218.97, -86.09] *	-0.04 [-0.21, 0.14]
1	-182.24 [-244.02, -120.45] *	-0.14 [-0.47, 0.19]
2	-170.22 [-229.89, -110.55] *	-0.20 [-0.63, 0.23]
3	-169.31 [-205.83, -132.79] *	-0.31 [-0.95, 0.33]
4	-149.37 [-250.41, -48.32] *	-0.36 [-1.08, 0.37]
5	-152.85 [-247.37, -58.34] *	-0.37 [-0.93, 0.19]
6	-185.19 [-216.12, -154.27] *	-0.24 [-0.57, 0.08]
7	-194.11 [-234.45, -153.77] *	-0.28 [-0.60, 0.04]

ß	-176.32 [-217.17, -135.47] *	-0.27 [-0.58, 0.05]
9	-175.63 [-216.99, -134.26] *	-0.25 [-0.59, 0.09]
10	-182.20 [-238.21, -126.19] *	-0.30 [-0.73, 0.12]
11	-168.77 [-242.16, -95.38] *	-0.49 [-1.19, 0.21]
12	-179.55 [-225.94, -133.16] *	-0.45 [-1.05, 0.15]

Supplemental Figure 5. Effect of Opting out of SNAP-EA on Monthly Benefit Size by Opt-Out Month

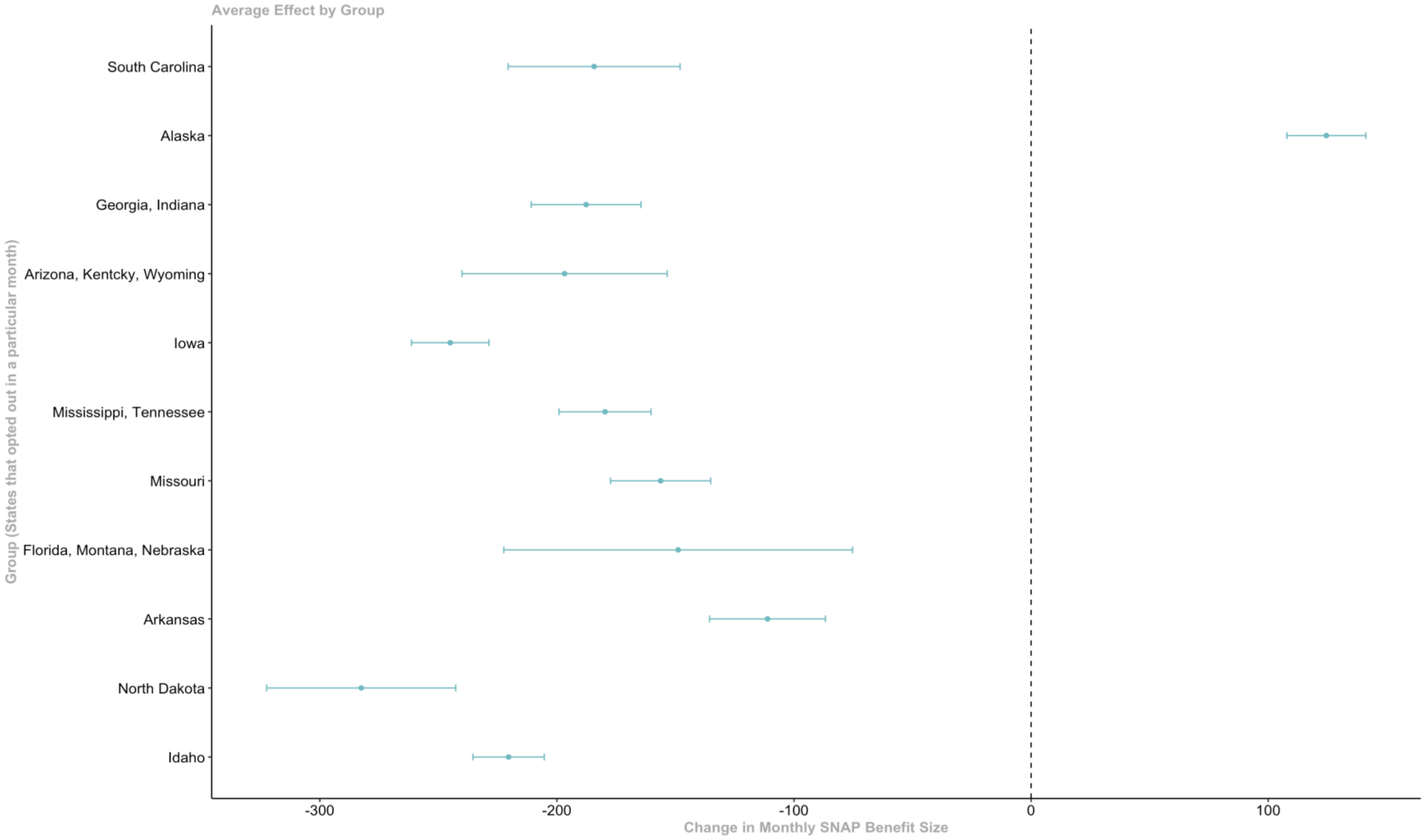

Supplemental Figure 6. State-Level Trajectories in SNAP Household Benefit Size after opting out of SNAP-EA

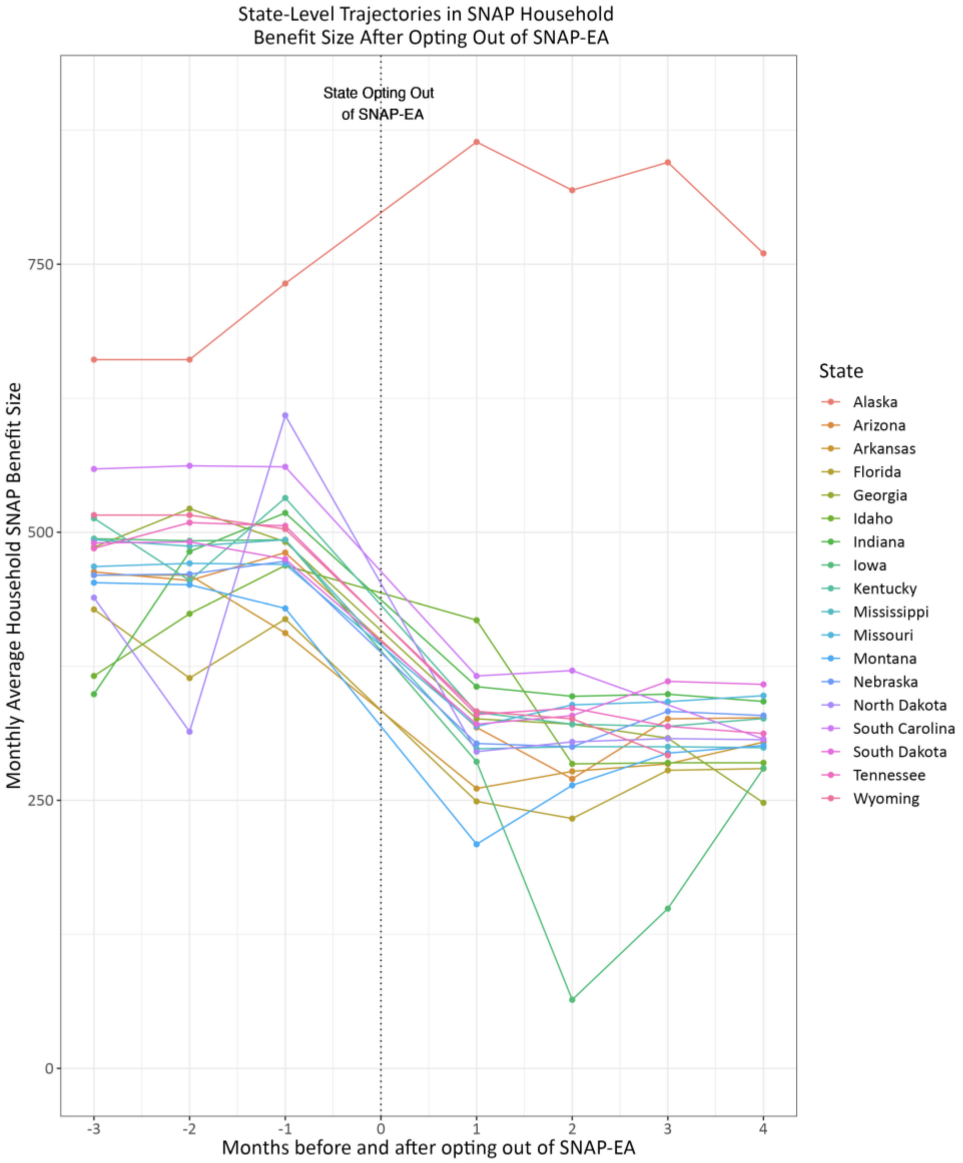

Supplemental Figure 7. Effect of Opting out of SNAP-EA on Percentage of Population Enrolled by Opt-Out Month

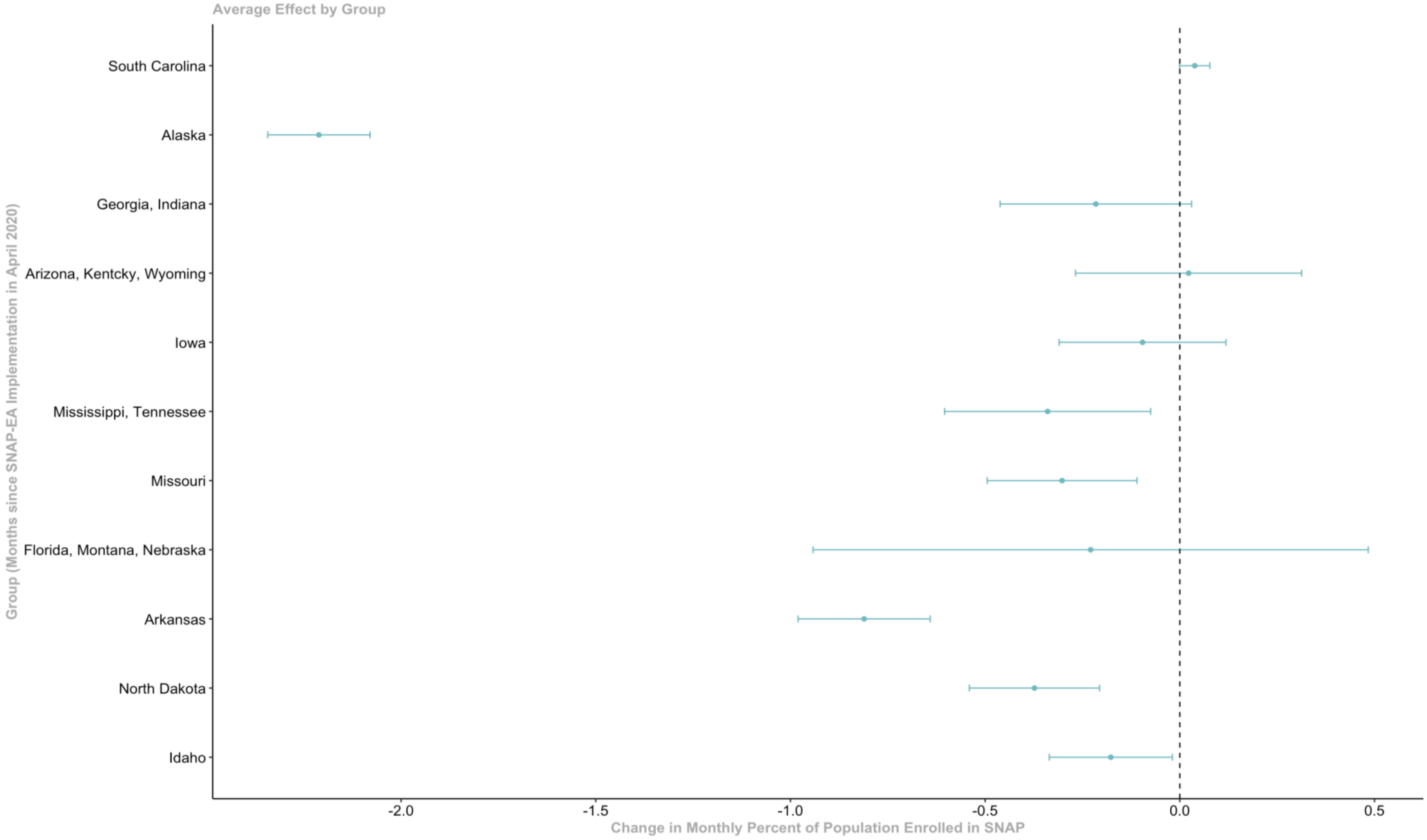

Supplemental Figure 8. State-Level Trajectories in Percentage of State Population Enrolled in SNAP after opting out of SNAP-EA

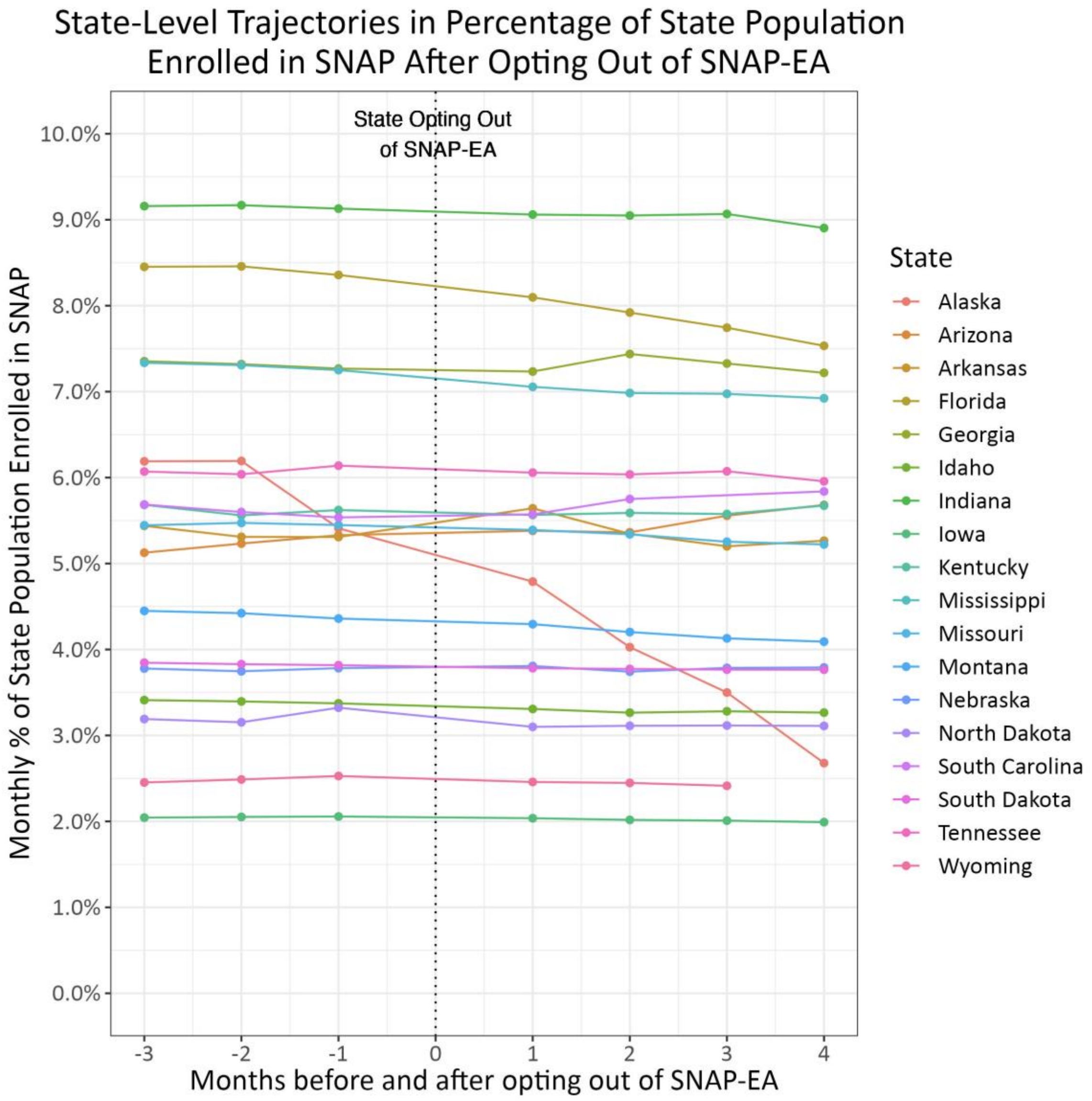

Supplemental Table 2. Average Effects of opting out of SNAP-EA by months before and after opt-out, adjusted for Republican governor

Average treatment effects	Average monthly SNAP benefit size	Percentage of state population enrolled in SNAP
**Group-time aggregate across all lengths of exposure, Adjusted for Republican Governor**	-184.33 [-218.22, -150.44] *	-0.49 [-0.90, -0.08] *
**Average Effect by Length of Exposure, Adjusted for Republican Governor**		
-12	12.69 [-80.57, 105.95]	-0.14 [-0.45, 0.18]
-11	-19.57 [-72.58, 33.43]	0.05 [-0.16, 0.26]
-10	-0.08 [-21.79, 21.63]	-0.05 [-0.25, 0.16]
-9	18.69 [-47.98, 85.37]	-0.10 [-0.31, 0.10]
-8	-5.22 [-64.60, 54.15]	-0.02 [-0.18, 0.14]
-7	-16.19 [-42.31, 9.94]	-0.09 [-0.19, 0.01]
-6	-4.88 [-33.40, 23.64]	-0.01 [-0.12, 0.11]
-5	9.14 [-30.20, 48.48]	-0.01 [-0.11, 0.08]
-4	5.32 [-18.25, 28.89]	-0.01 [-0.09, 0.07]
-3	-20.65 [-52.53, 11.22]	0.00 [-0.07, 0.08]
-2	2.18 [-35.59, 39.95]	-0.03 [-0.14, 0.08]
-1	23.88 [-34.67, 82.43]	0.01 [-0.20, 0.22]
0	-150.19 [-218.06, -82.33] *	-0.08 [-0.23, 0.07]
1	-179.56 [-249.12, -110] *	-0.20 [-0.52, 0.13]
2	-174.82 [-230.14, -119.50] *	-0.30 [-0.69, 0.1]
3	-170.7 [-213.46, -127.94] *	-0.42 [-0.98, 0.14]
4	-154.43 [-258.64, -50.22] *	-0.45 [-1.12, 0.22]

5	-155.79 [-257.07, -54.51] *	-0.47 [-1.00, 0.06]
6	-186.22 [-219.88, -152.56] *	-0.36 [-0.78, 0.05]
7	-199.65 [-245.44, -153.87] *	-0.41 [-0.83, 0.01]
8	-176.87 [-218.11, -135.63] *	-0.37 [-0.81, 0.07]
9	-177.65 [-219.81, -135.48] *	-0.37 [-0.89, 0.16]
10	-177.47 [-231.68, -123.26] *	-0.44 [-1.10, 0.23]
11	-168.17 [-242.36, -93.99] *	-0.65 [-1.55, 0.26]
12	-185.33 [-232.99, -137.67] *	-0.61 [-1.42, 0.19]

Supplemental Table 3. State-Level Opt-out Regression Covariate Summary Statistics

**Variable**	**States that Remained in SNAP-EA (N = 33)**	**States that Opted Out of SNAP-EA (N = 18)**
Republican governor from 2021-2023 N (%)	10 (30.3%)	17 (94.4%)
2022 PVI Mean ± SD (Range)	-2.30 ± 12.25 (-43, 22)	11.72 ± 6.22 (2, 25)
Population (2019, millions) Mean ± SD (Range)	7.35 ± 8.28 (0.62, 39.5)	4.75 ± 5.05 (0.58, 21.5)
Unemployment rate (2019) Mean ± SD (Range)	3.64 ± 0.75 (2.4, 5.5)	3.59 ± 0.96 (2.4, 6.1)
SNAP Policy Index (April 2020, weighted) Mean ± SD (Range)	7.57 ± 1.51 (3.61, 8.83)	5.78 ± 1.46 (2.37, 8.48)
